# Furanoids from the *Gymnadenia conopsea* (Orchidaceae) seed germination supporting fungus *Ceratobasidium* sp. (GS2)

**DOI:** 10.3389/fmicb.2022.1037292

**Published:** 2022-11-17

**Authors:** Lixin Shi, Li Han, Zeyu Zhao, Qi Li, Yanduo Wang, Gang Ding, Xiaoke Xing

**Affiliations:** ^1^Key Laboratory of Bioactive Substances and Resources Utilization of Chinese Herbal Medicine, Ministry of Education, Institute of Medicinal Plant Development, Chinese Academy of Medical Sciences and Peking Union Medical College, Beijing, China; ^2^Institute of Microbiology, Chinese Academy of Sciences, Beijing, China

**Keywords:** furanoids, symbiotic fungus, *Ceratobasidium* sp., antioxidant activity, phytotoxicity assay, germination assay

## Abstract

Five furanoids including a new analog (*S*)-1,4-di(furan-2-yl)-2-hydroxybutane-1,4-dione (**1**) together with four known ones, rhizosolaniol (**2**), 5-hydroxymethylfurfural (**3**), 2-furoic acid (**4**) and (2-furyl) oxoacetamide (**5**), were isolated from the fungal strain *Ceratobasidium* sp. (GS2) inducing seed germination of the endangered medicinal plant *Gymnadenia conopsea* of Orchidaceae. The structure of new furanoid **1** was determined mainly based on HR-ESI-MS and NMR spectral data. Modified Mosher’s reactions were used to establish the stereochemistry of the hydroxyl group in **1**, which was not stable in Mosher’s reagents and transformed into four analogs **6**–**9**. These degraded products (**6**–**9**) were elucidated based on UPLC-Q-TOF-MS/MS analysis, and compound **8** was further isolated from the degraded mixture and its structure was characterized through NMR experiments. Therefore, the absolute configuration of compound **1** was determined by electronic circular dichroism combined with quantum-chemical calculations adopting time-dependent density functional theory. Compounds (**1**–**5**), and **8** showed weak antioxidant activities, and compounds (**2**–**4**) displayed phytotoxicity on punctured detached green foxtail leaves. In addition, compounds **3** and **4** strongly showed inhibition activities on the seed germination of *G. conopsea*. This was the first chemical investigation of the symbiotic fungus of *G. conopsea*.

## Introduction

The Orchidaceae is one of the largest plant families with estimated 26,000 species (Jacquemyn et al., 2011). Most orchid species are dependent on mycorrhizal fungi to supply nutrients for seed germination due to their tiny seeds devoid of endosperm **(**Bidartondo and Read, 2008; Xing et al., 2015; Rafter et al., 2016). The fungi possess the ability to supporting seed germination mainly including members of *Ceratobasidiaceae*, *Tuleasnellaceae*, *Serependiaceae*, and a few *Ascomycetes* (Rafter et al., 2016). These fungi contain different chemical constituents, such as protoilludane sesquiterpenoid aromatic esters, diterpenoids, triterpenoids, polysaccharides, and adenosines, which have diverse biological activities (Wang et al., 1992).

**Figure 1:**
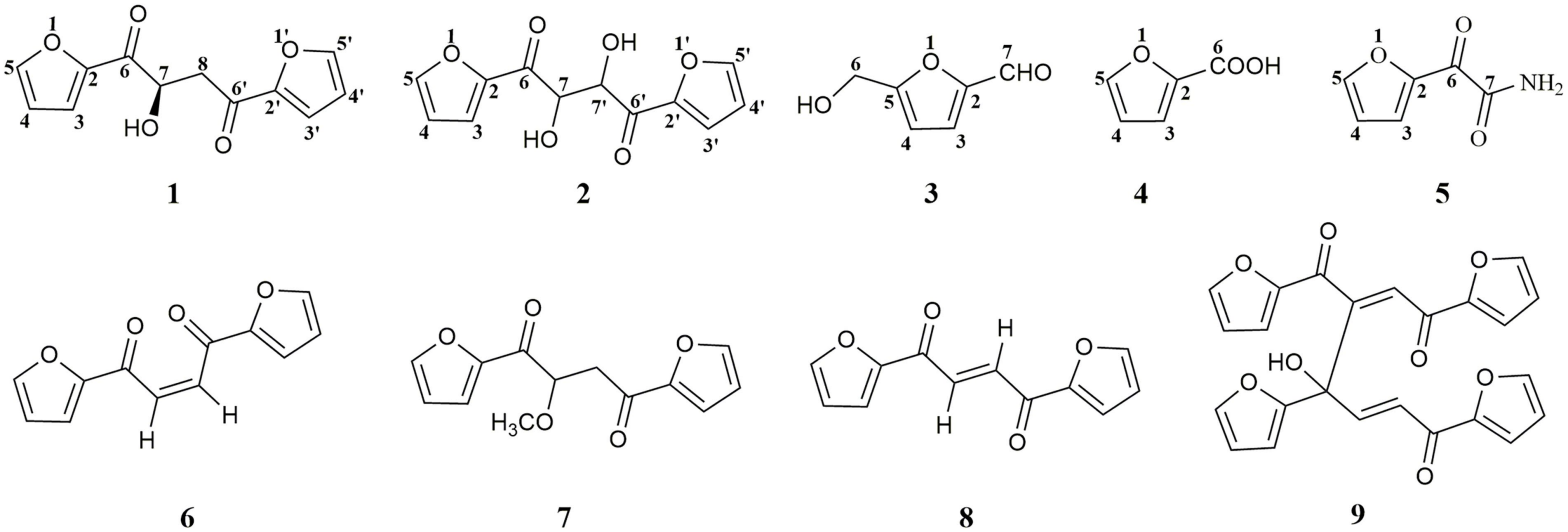
The structures of the compounds **1**–**9.**

*Gymnadenia conopsea* (L.) R. Br. is a perennial herbaceous orchid plant that grows widely throughout Europe and in temperate and subtropical zones of Asia (Stark et al., 2009). In China, tuber of *G. conopsea* has been used in traditional Chinese medicines, Tibetan medicines, Mongolian medicines, and other ethnic medicines (Shang et al., 2017). However, this species has suffered dramatic declines in distribution and abundance due to habitat loss, overgrazing, and over-collection in recent years. In order to realize the sustainable utilization of *G*. *conopsea* resource, an effective way was to obtain the fungal strain with the ability to support seed germination and further used in recovery of wild populations. One fungal strain GS2, initially isolated from roots of *G*. *conopsea*, was able to support *G*. *conopsea* seeds germination under both artificial and natural conditions (Gao et al., 2019, 2020; Jiang et al., 2022). The fungal strain GS2 was identified as *Ceratobasidium* sp. (Gao et al., 2020). Some secondary metabolites of the symbiotic fungus may play a role in the interaction between host plants and fungi. To date, few studies on the chemical constituents of Ceratobasidiaceae fungi have been carried out, and chemical composition of the fungal strain GS2 has never been investigated. During the process of finding the new and bioactive secondary metabolites from symbiotic fungi (Ding et al., 2014; Song et al., 2019; Zhang et al., 2019, 2020), five furanoids analogs (**1**–**5**) were isolated from GS2 and further identified. Interestingly, when compound **1** was added into Mosher’s reagents, four other analogs (**6**–**9**) were formed, implying that the structure of **1** was not stable in the solvent of Mosher’s reagents. In this report, the isolation, structural elucidation, and biological evaluations of furanoids **1**–**5**, and structural analysis of degraded products (**6**–**9**) were present (Figure 1).

## Materials and methods

### General experimental procedures

All ^1^H and ^13^C-NMR data were measured on a Bruker 600 spectrometer operating at 600 (^1^H) and 150 (^13^C) MHz. HR-ESI-MS were obtained using a UPLC-Q-TOF-MS/MS (Waters Synapt G2, United States). Optical rotations were measured on a 241 polarimeter (PerkinElmer, Waltham, United States). CD spectra were recorded with a JASCO J-815 spectropolarimeter. UV-2102 (Unico, Shanghai, China) was used to measure UV data. IR data were acquired using an FTIR-8400S spectrophotometer (Shimadzu, Kyoto, Japan). Semi-preparative HPLC separation was performed on a Shimadzu LC-6 AD instrument packed with a YMC-Pack ODS-A column (5 μm, 250 × 10 mm). Sephadex LH-20 (Pharmacia Biotech, Sweden) and silica gel (60–100, 100–200 mesh; Qingdao Marine Chemical Factory, Qingdao, China) were used for column chromatography.

### Fungal strain and fermentation

**Figure 2:**
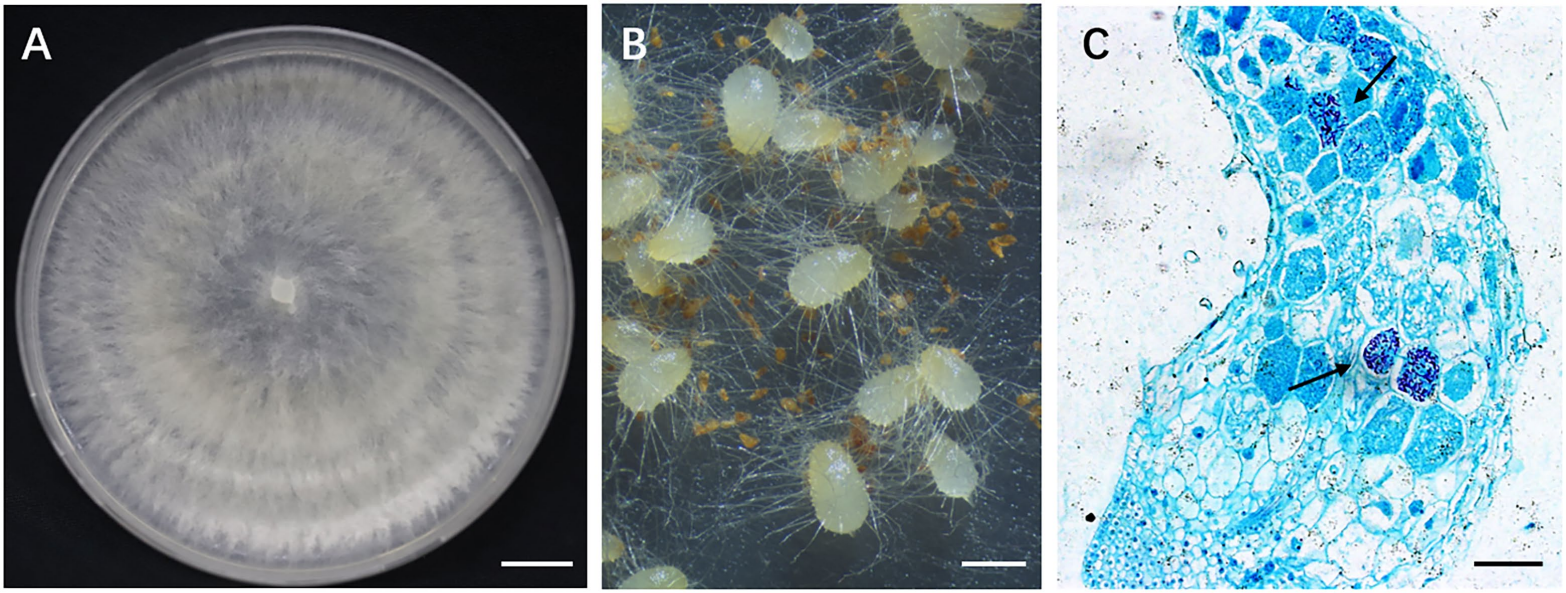
*Gymnadenia conopsea* seed germination promoted by GS2. **(A)** Colony morphology of GS2; **(B)** germinating seeds of *G. conopsea*, protocorms formed 30 days after incubated with inoculum of GS2; **(C)** cross section of protocorm. Large number of pelotons (arrow) were observed in cortical cells (arrow). Bar A = 1 cm; B = 1 mm; C = 200 μm.

The fungal strain GS2 (NCBI accession no.: OK655751.1) was previously isolated from the roots of *G. conopsea* and shown to promote seed germination and early seedling development under *in vitro* conditions (Gao et al., 2020). The strain was deposited in the Mycological Herbarium of the Institute of Microbiology, Chinese Academy of Sciences (accession number CGMCC no. 16089). The fungus was cultured on potato dextrose agar (PDA) in 9 cm Petri dishes at 23°C in dark. After the colony had reached a diameter of 8 cm, the plates were used for inoculation (Figure 2). Two 1 cm × 1 cm plugs of fungal strain taken from fresh cultures was inoculated into the sterilized solid medium with the formula of rice (60.0 g) and distilled water (80 ml) in 250 conical flasks (500 ml) for further fermentation at room temperature for 40 days.

### Extraction and isolation

The fermented material was extracted with ethyl acetate for 3 times and soaked for 72 h each time, and the organic solvent was evaporated to dryness under vacuum to afford 100.0 g of extract. The crude extract (100.0 g) was subjected to a silica gel column chromatography (CC) eluting with petroleum ether-acetone (100:0, 100:1, 50:1, 20:1, 10:1, 5:1, 2:1, 1:1 and 0:1, v/v) to obtain seven fractions (Fr.1-Fr.7). Fr.4 (647 mg) was separated by a silica gel CC eluted with petroleum ether- acetone (100: 1, 50:1, 20: 1, 10:1, 5:1, 2:1, and 0:1, v/v) to get 5 subfractions (Fr.4.1–Fr.4.5). Fr.4.5 (49 mg) was then purified by semi-preparative HPLC (UV 210 nm and 254 nm, 40–60% CH_3_OH-H_2_O for 30 min, v/v, 2 ml/min) to afford **5** (2 mg, *t*_R_ = 10 min). Fr.5 (3.88 g) was separated by a silica gel CC eluted with petroleum ether-acetone (100: 1, 50:1, 20: 1, 10:1, 5:1, 2:1, and 0:1, v/v) to get 6 subfractions (Fr5.1–Fr5.6). Fr5.6 (82 mg) was then purified by semi-preparative HPLC (UV 210 nm and 254 nm, 20–40% CH_3_OH-H_2_O for 30 min, v/v, 2 ml/min) to afford **2** (4 mg, *t*_R_ = 15 min) and **1** (6 mg, *t*_R_ = 18 min). Fr.6 (1 g) was separated by a silica gel CC eluted with petroleum ether- acetone (100: 1, 50:1, 20: 1, 10:1, 5:1, 2:1, and 0:1, v/v) to get 4 subfractions (Fr.6.1–Fr.6.4). Fr.6.2 (203 mg) was then purified by semi-preparative HPLC (UV 210 nm and 254 nm, 30–100% CH_3_OH-H_2_O for 30 min, v/v, 2 ml/min) to afford **3** (8 mg, *t*_R_ = 15 min) and **4** (15 mg, *t*_R_ = 19 min).

(*S*)-1,4-di(furan-2-yl)-2-hydroxybutane-1,4-dione (**1**): [*α*]D^25^ + 20.0 (*c* 0.1, MeOH); UV (MeOH) *λ*_max_ (log *ε*) 224 (4.48) nm (Supplementary Figure S1); CD (*c* 0.68 μM, MeOH): *λ*(Δ*ε*) 250 (0.65), 290 (−0.40); IR (neat) ν_max_ 3,444, 3,134, 1,668, 1,569, 1,466, 1,395, 1,288, 1,228, 1,162, 1,083, 1,033, 904, 883, 844, 769, 593 cm^−1^ (Supplementary Figure S2); ^1^H-NMR and ^13^C-NMR data see Table 1.

**Table 1:** ^1^H (600 MHz) and ^13^C (150 MHz) NMR for 1 in DMSO-*d*_6_.

Pos.	**1**	
	*δ*_C,_ type	*δ*_H_ (*J* in Hz)
2	150.4, C	
3	120.2 (overlapped), CH	7.56, d, (3.6)
4	112.7, CH	6.73, dd, (1.8, 3.6)
5/5’	148.2, CH	8.04, d, (1.8)
6	188.2, C	
7	69.3, CH	5.10, dt, (6.6, 7.2)
8	42.2, CH_2_	3.22, t, (6.6)
2’	152.0, C	
3’	119.2, CH	7.50, d, (3.6)
4’	112.8, CH	6.74, dd, (1.8, 3.6)
6’	185.8, C	
7-OH		5.85, d, (6.6)

Rhizosolaniol (**2**): ^1^H-NMR ((CD_3_)_2_CO, 600 MHz) *δ*_H_ = 8.01 (2H, dd, *J* = 1.8, 0.6 Hz, H-5, H-5′), 7.61 (2H, dd, *J* = 3.6, 0.6 Hz, H-2, H-2′), 6.79 (2H, dd, *J* = 3.6, 1.8 Hz, H-4, H-4′), 5.40 (2H, s, 7-OH, 7′-OH).

5-hydroxymethylfurfural (**3**): ^1^H-NMR (CDCl_3_, 600 MHz) *δ*_H_ = 9.61 (1H, s, CHO), 7.22 (1H, d, *J* = 3.6 Hz, H-3), 6.53 (1H, d, *J* = 3.6 Hz, H-4), 4.73 (1H, s, H-6).

2-furoic acid (**4**): ^1^H-NMR (DMSO-*d*_6_, 600 MHz) *δ*_H_ = 7.08 (1H, d, *J* = 1.8 Hz, H-5), 7.05 (1H, d, *J* = 3.6 Hz, H-3), 6.58 (1H, dd, *J* = 3.6, 1.8 Hz, H-4).

(2-furyl) oxoacetamide (**5**): ^1^H-NMR (CDCl_3_, 600 MHz) *δ*_H_ = 8.15 (1H, d, *J* = 3.6 Hz, H-5), 7.78 (1H, d, *J* = 1.8 Hz, H-3), 6.64 (1H, dd, *J* = 1.8, 3.6 Hz, H-4), 0.98(1H, s, -NH), 0.81(1H, s, -NH).

2,3-dihydroxy-1,4-furan-2-yl-1,4-butanedione (**8**): ^1^H-NMR (CDCl_3_, 600 MHz) *δ*_H_ = 7.79 (2H, s, H-7, H-7′), 7.73 (2H, d, *J* = 1.8 Hz, H-5, H-5′), 7.45 (2H, d, *J* = 3.6 Hz, H-3, H-3′), 6.65 (2H, dd, *J* = 3.6, 1.8 Hz, H-4, H-4′).

### Modified Mosher’s reaction

The powder of **1** (2.0 mg) was transferred to two small clean bottles, respectively, and then pyridine-*d*_5_ (0.5 ml) and (*R*)-MTPACl/(*S*)-MTPACl (8.0 μl) were quickly added into the clean bottles. All contents were mixed thoroughly by shaking the clean bottles carefully. The reaction was performed at room temperature, and the solution was allowed to stand for 24 h. The modified Mosher’s reaction products (*R* and *S*) were analyzed through HPLC experiments (Lumtech; YMC-Pack ODS-A column; 10 μm; 250 × 10 mm; 2 ml/min, 75% MeOH in H_2_O for 30 min), and four peaks (**6**–**9**) were observed, in which the main transformed product (**8**) was purified by semi-preparative HPLC (UV 210 nm and 254 nm, 30–100% CH_3_OH-H_2_O for 30 min, v/v, 2 ml/min, *t*_R_ = 27 min).

### UPLC-MS/MS analysis

Compounds **1** and **6**–**9** were analyzed using a UPLC-Q-TOF-MS/MS system (Waters, United States). Chromatographic analysis was carried out with a Waters Acquity UPLC-PDA system combined with an analytical reverse-phase C-18 column (2.1 × 100 mm, 1.7 μm, Acquity BEH, Waters, United States), and the detection wavelength of the PDA detector was 200 to 400 nm. The column temperature was set at 40°C. As the mobile phase, 0.1% formic acid in water (A) and 0.1% formic acid in acetonitrile (B) were used. The gradient conditions were as follows: 0–2 min, 10% B; 2–10 min, 10–80% B; 10–13 min, 80–100% B; 13–15 min, 100%; and 15–17 min, 10% B. The flow rate from the UPLC system into the UPLC-Q-TOF-MS/MS detector was 0.3 ml/min. The injection volume was 0.3 μl. Time-of-flight MS detection was performed with a Waters SYNAPT G2 HDMS (Waters Corp., Manchester, UK) TOF mass spectrometer equipped with an ESI source in positive ion scan mode. The source temperature was 80°C, and the desolvation temperature was maintained at 450°C with desolvation gas flow at 900.0 l/h. The lock mass in all analyses was leucine-enkephalin ([M + H]^+^ = 556.2771), used at a concentration of 200 μg/ml and infused at a flow rate of 10 l/min. Raw data was acquired using the centroid mode and the mass range was set from *m/z* 100 to 1,000. The capillary voltage was set at 2.5 kV with 30 V of sample cone voltage. The collision energy was set as 6 eV for low-energy scan and a ramp from 30 to 50 eV for the high-energy scan. The instrument was controlled by MassLynx 4.1 software.

### Antioxidant activities

The ability of scavenging free radicals of six compounds were assessed by reacting with the 2,2-diphenyl-1-picrylhydrazyl (DPPH) (Salar et al., 2017; Fu et al., 2022). The compounds and Vitamin E were used to prepare concentrations of 5, 10, 20, 40, 60, 80, 100 and 200 μg/ml in methanol. The DPPH powder (0.8 mg) was dissolved in methanol in a 20 ml brown volumetric flask before mixed with samples in ratios of 1:1. After 3 h of incubation in the dark, the absorbance was taken in the microplate format at 517 nm. The scavenging efficiency was compared with Vitamin E as a basic and methanol solution as control.

The scavenging rate of DPPH according to the following formula:

(1) $$\mathrm{Scavenging}\;\mathrm{rate}\;\left(\%\right)=\frac{\left(A_{0}-A_{1}\right)\;}{A_{0}}\times 100$$

*A*_0_: the absorbance value of the control group; *A*_1_: the absorbance value of the experimental group.

### Phytotoxicity assay

The phytotoxicities of pure compounds **1**–**5** on green foxtail were tested by using leaf-puncture assay. The fresh leaves were cut into segments approximately 4 cm long to be placed on a glass slide in a sterile petri dish with one layer of absorbent paper wetted with sterile water. Filter paper disks of 5 mm diameter were prepared using a perforator and sterilized at 121°C for 30 min. A 1 mg amount of test compounds was dissolved in DMSO to produce the 1 mg/ml test solution. In the leaf-puncture assay, a small mild lesion was made from each other on each leaf segment with a nipper and covered with a filter paper disks. Then 20 μl of DMSO or 20 μl of test solution was added to each disk. All experiments were repeated at least three times. The effects were recorded after 3 days of observation (Zhao et al., 2021).

### Germination assay

*G. conopsea* seeds were surface sterilized by 5% sodium hypochlorite for 5 min, followed by washing with sterile distilled water five times. Compounds **3** and **4** were dissolved with 0.5 ml DMSO. Then they were added to 15 ml of PDA medium supplemented with 0.8% (w/v) agar. Finally, the concentrations of the compounds in the medium were 0.01, 0.1, 1 mg/ml, respectively. To eliminate the effect of DMSO, plates with 0.5 ml DMSO were used as blank control. Seeds were distributed on each Petri dish as described above. A fresh mycelium (GS2) was also placed in each plate because the seeds of *G. conopsea* could not grow in the absence of the fungus. Each concentration was conducted in triplicate. The Petri dishes were placed in dark room at 25°C for 45 days. The growth of fungus and the number of seeds germination were recorded.

## Results and discussion

### Structural identification

**Figure 3:**
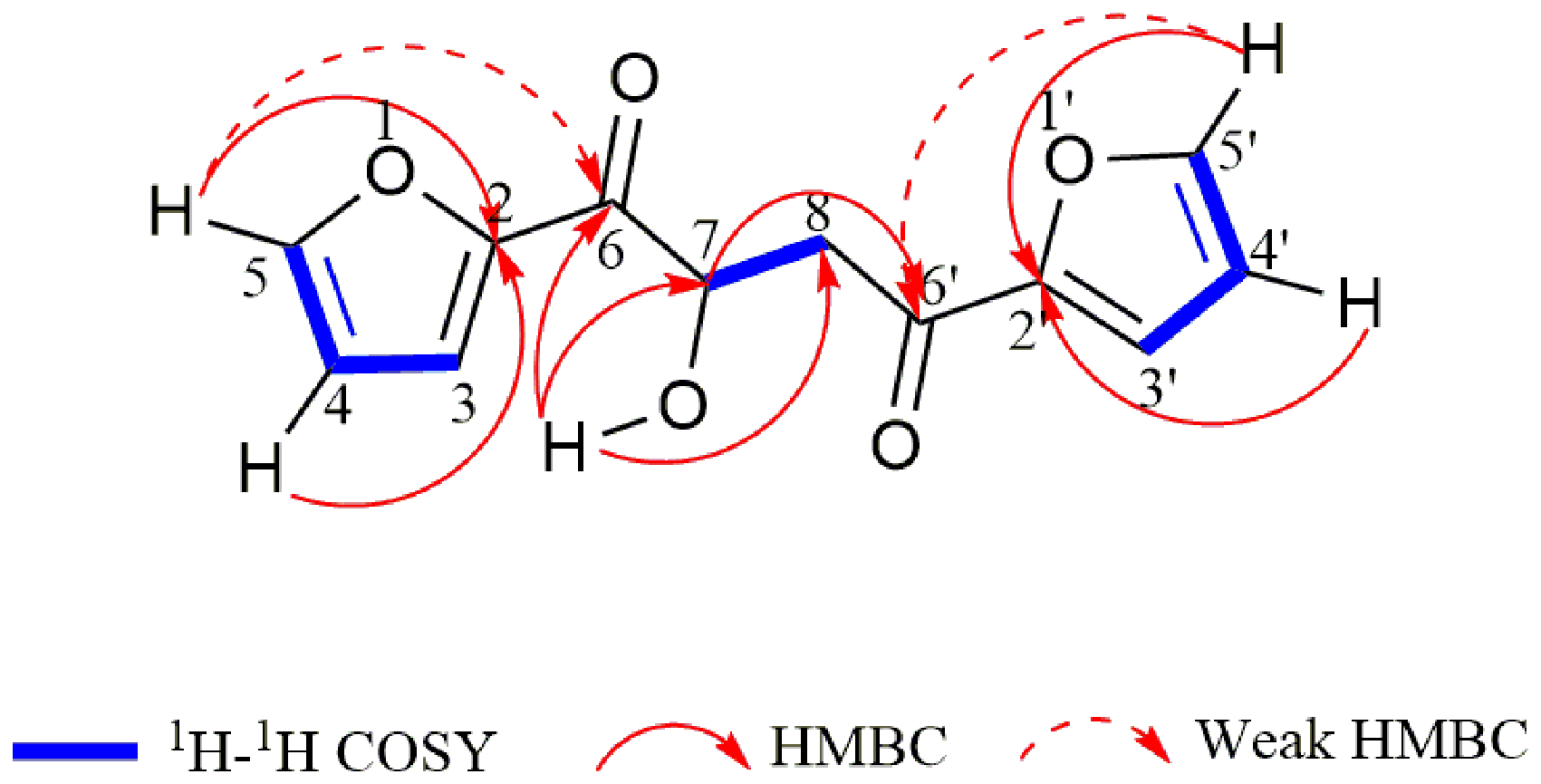
^1^H-^1^H COSY and HMBC correlations of compound **1**.

Compound **1** was obtained as a pale yellowish oil, and its molecular formula was assigned as C_12_H_10_O_5_ based on HR-ESI-MS data (Supplementary Figure S3; *m/z* 257.0426 [M + Na]^+^) with eight degrees of unsaturation. The IR spectrum showed absorption bands at 3444 and 1,668 cm^−1^, which implied the presence of hydroxyl and carbonyl groups. Analysis of the ^1^H-NMR, ^13^C-NMR (Table 1) and HSQC data (Supplementary Figures S4–S8) for **1** revealed the presence of a methylene, an oxygenated methine, a free hydroxyl proton, two carboxyl carbons and eight olefinic carbons (six of them were protonated). The planar structure of **1** was elucidated by extensive analysis of its ^1^H-^1^H COSY and HMBC spectral data (Figure 3). The ^1^H-^1^H COSY correlations gave three isolated proton spin systems corresponding to C-3-C-4-C-5, C-3′-C-4′-C-5′ and C-7-C-8, and the remaining connections were characterized through HMBC spectrum. The HMBC correlations from H-5 to C-2, from H-4 to C-2, from H-5′ to C-2′ and from H-4′ to C-2′ combined with the chemical shift of C-5/5′ (148.5) and C-2/2′ (152.0) indicated that there was an oxygen atom between C-5/5′ and C-2/2′ to form two furan rings (Yang et al., 2010); the weak long-ranged correlations from H-5 to C-6 and H-5′ to C-6′ confirmed the connectivities from C-2 to C-6, C-2′ and C-6′; the correlations from OH-7 to C-6, C-7, C-8, from H-7 and -CH_2_-8 to C-6′ determined the direct connectivities of C-7/C-6, and C-8/C-6′ with a hydroxyl group anchored at C-7. Thus, the planar structure of **1** was established as a new furanoid analog named 1,4-di(furan-2-yl)-2-hydroxybutane-1,4-dione.

Modified Mosher’s method was used to determine the absolute configuration of secondary hydroxyl group in different small molecules (Tran et al., 2020; Fu et al., 2021). As **1** possessed a secondary hydroxyl at C-7, modified Mosher’s reaction was tried to determine its stereochemistry, whereas the reaction was not successful. After compound **1** was mixed with Mosher’s reagents, four main products (4 peaks were observed in the HPLC experiments) were produced, indicating that compound **1** was not stable in the solvent of Mosher’s reagents. To clarify the transformation process, the products were then analyzed based on UPLC-MS/MS experiments.

**Figure 4:**
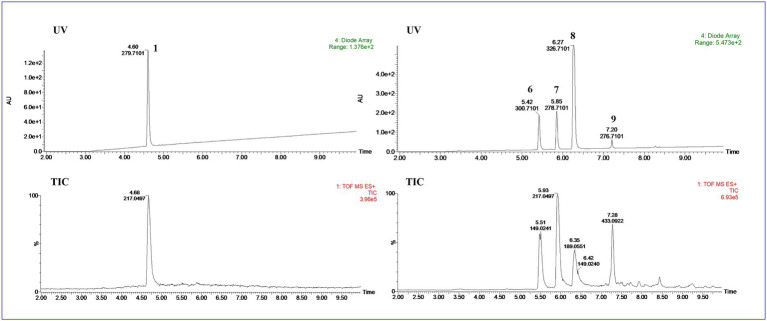
The UPLC-Q-TOF-MS UV and total ion chromatogram (TIC) of compound (**1**) and degraded products (**6**–**9**) in modified Mosher’s regents.

**Figure 5:**
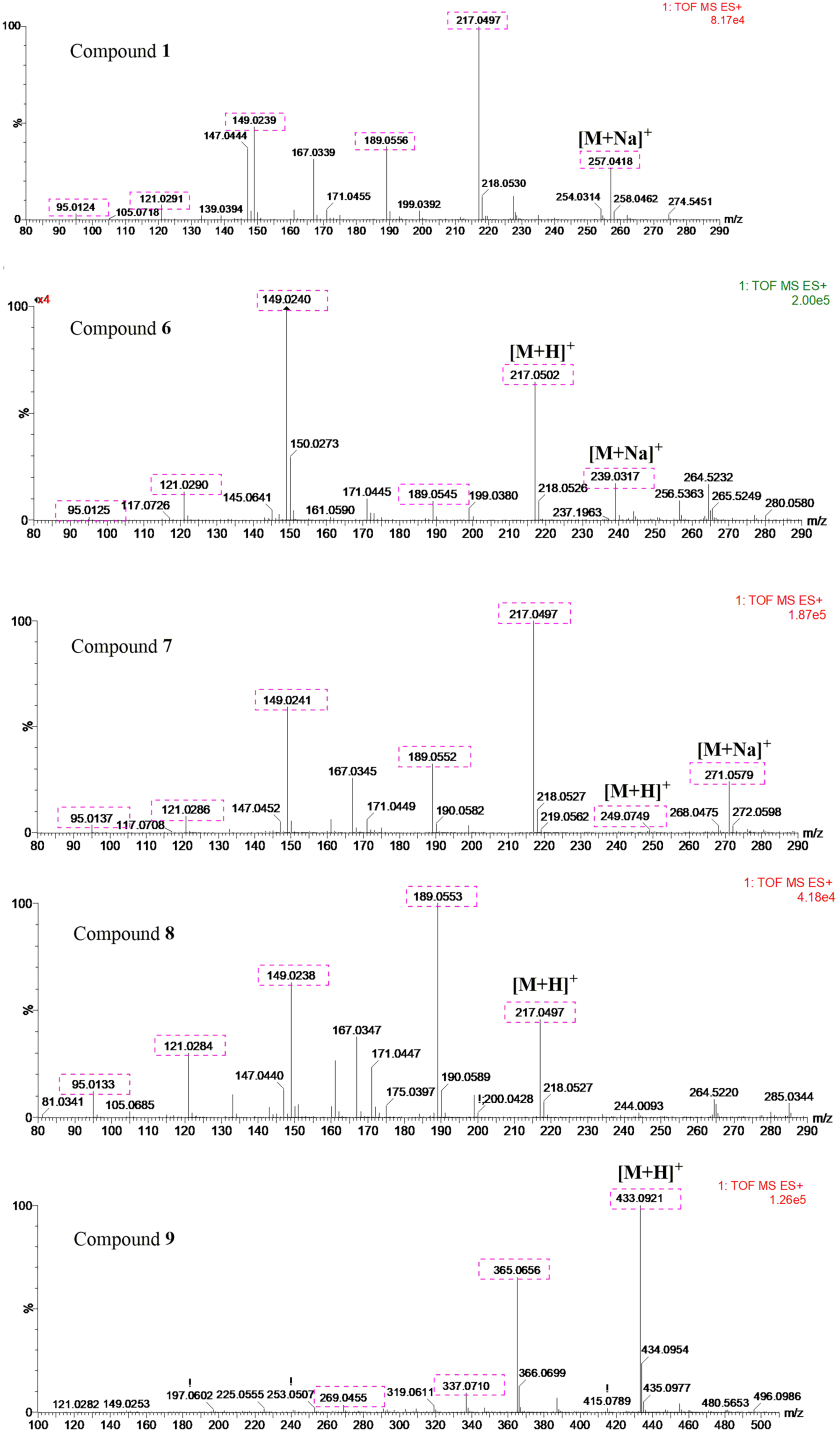
The analysis of UPLC-Q-TOF-MS spectra of compounds **1** and **6–9**.

**SCHEME 1:**
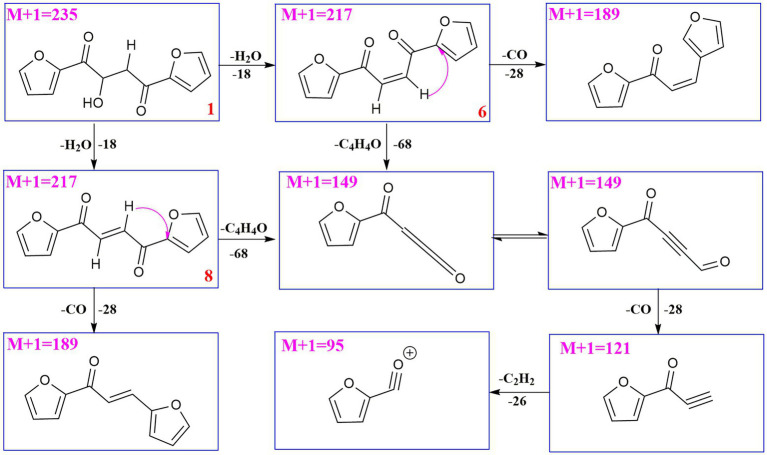
Possible fragmentation pathways of compound **1**.

UPLC-MS/MS analysis (Figures 4, 5) revealed that compound **1** had transformed into four analogs **6**–**9** after mixed with Mosher’s reagents (deuterated pyridine as solvent). Based on high resolution mass and fragmentation mechanisms of the transformed products, the possible structures of **6**–**9** were tentatively elucidated. The high-resolution mass and fragment ions together with the elemental constituents were listed in Supplementary Tables S1–S5. The fragmentation routes of compound **1** (*t*_R_ = 4.68 min) according to UPLC-Q-TOF-MS/MS analysis was depicted in Scheme 1, in which typical neutral losses and 1,3-rearrangement were the main fragmentation patterns. The parent ion (*m/z* 249) of **1** was not observed, whereas ions (*m/z* 257, [M + Na]^+^) were present in the UPLC-Q-TOF-MS/MS spectrum. This might be because the parent ion (*m/z* 249) of **1** was extremely unstable and easily lost H_2_O molecule to produce daughter ions at *m/z* 217. Then, this ion (*m/z* 217) could form the ion (*m/z* 189) by neutral loss of CO or produced ion *m/z* 149 by 1,3-rearrangement, which yielded other product ions at *m/z* 121 and 95 through neutral loss of CO and C_2_H_2_.

**SCHEME 2:**
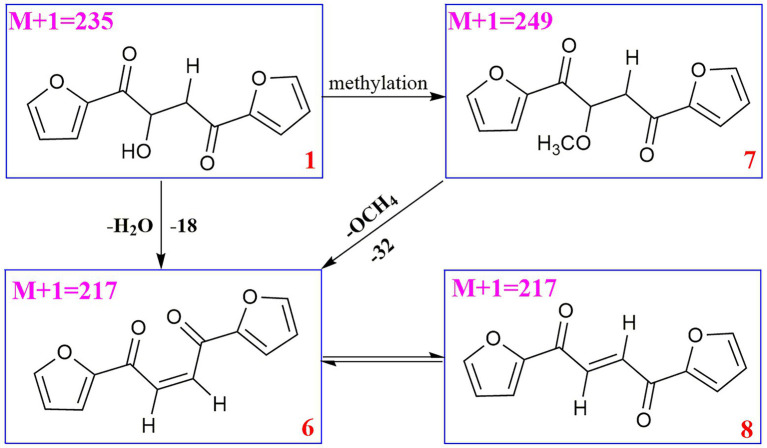
Possible fragmentation pathways of compounds **6–8**.

**SCHEME 3:**
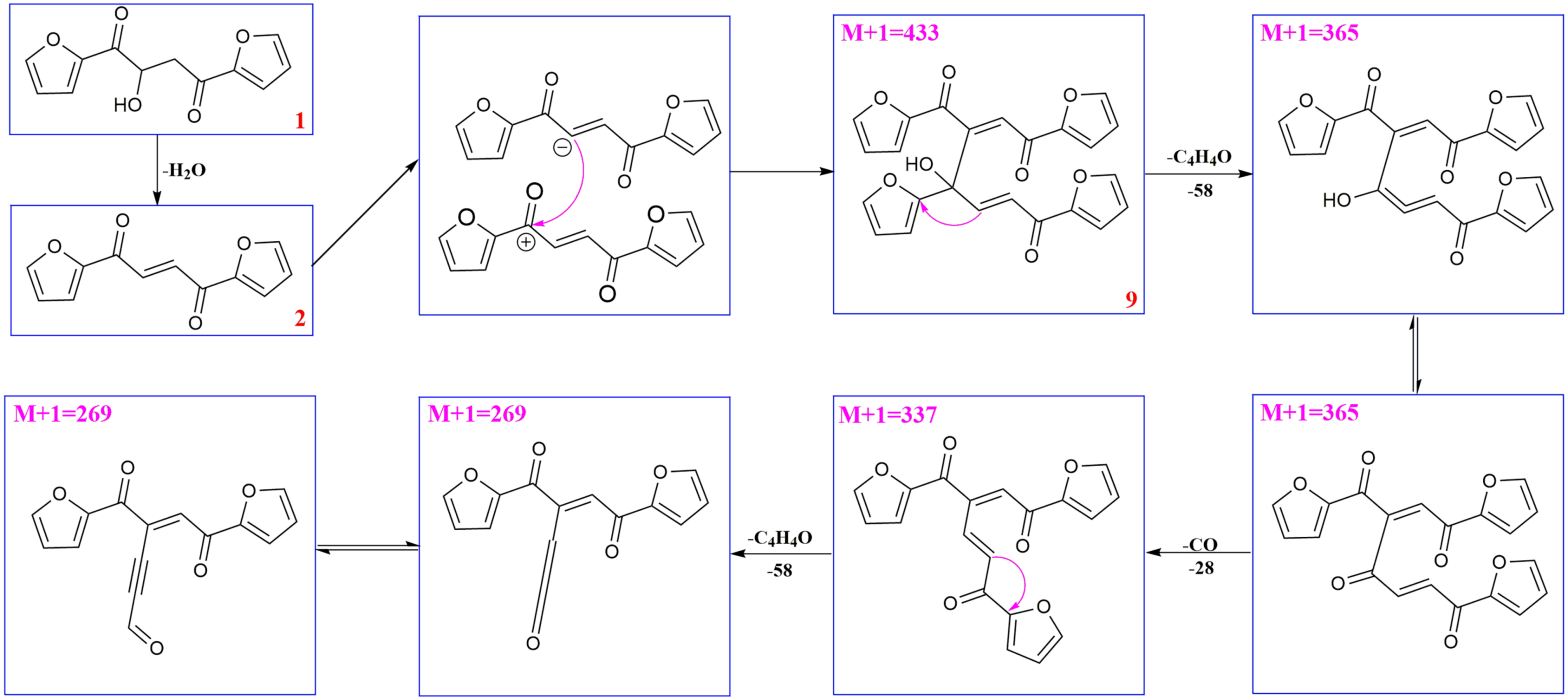
Possible fragmentation pathways of compound **9**.

Compound **6** (*m/z* 217, [M + H]^+^) was the product of dehydration of compound **1** based on the UPLC-Q-TOF-MS/MS profile, which retention time was 5.51 min (Scheme 2). It possessed the same fragmentation ions (*m/z* 189, 149, 121, and 95) as compound **1**. The [M + H]^+^ parent ion at *m/z* 249 and the [M + Na]^+^ parent ion at *m/z* 257 of **7** together with high resolution mass suggested its molecular formula as C_13_H_12_O_5_ was 14 Daltons more than that of **1**, implying that an additional methyl group was attached at the hydroxyl. The neutral loss of 32 Daltons to form the product ion *m/z* 217 confirmed this conclusion, and other daughter ions were same as those of **1**. Compounds **6** (*t*_R_ = 5.51 min) and **8** (*t*_R_ = 6.35 min) was a pair of isomers which had the same molecular formula and similar MS/MS profiles. The *cis*-double bond in structure of **6** led to the steric hindrance between the two carbonyls, which would lead to blue shift compared with **8** in UV spectra (Supplementary Figure S9), while the *trans*-double bond in structure of **8** led to the distance reduction of the two carbonyls spatial position and its polarity (Xu et al., 2022). Thus, the structure of **8** was more stable according to thermodynamic considerations (Abou-Gazar et al., 2004), which led to more field of **8** than that of **6**. An ion (*t*_R_ = 7.28 min) at *m/z* 433 ([M + H]^+^) was detected and its chemical formula was determined to be C_24_H_16_O_8_, indicating that compound **9** might be a dimer. The parent ion at *m/z* 433 easily lost furan rings and CO to produce daughter ion at *m/z* 269 (Scheme 3).

To further support the conclusion mentioned above, the main compound **8** of the transformed product was purified. The molecular formula of **8** was assigned as C_12_H_8_O_4_ based on HR-ESI-MS data (*m/z* 217.0497, [M + H]^+^; Supplementary Figures S10, S11), whereas only four protons were observed in the ^1^H-NMR spectrum, implying that this compound might be a symmetrical dimer. Based on comparison of the NMR data with those in reported in the literature, the structure of **8** was determined to be 2,3-dihydroxy-1,4-furan-2-yl-1,4-butanedione (Supplementary Figure S12; Wang et al., 2020).

**Figure 6:**
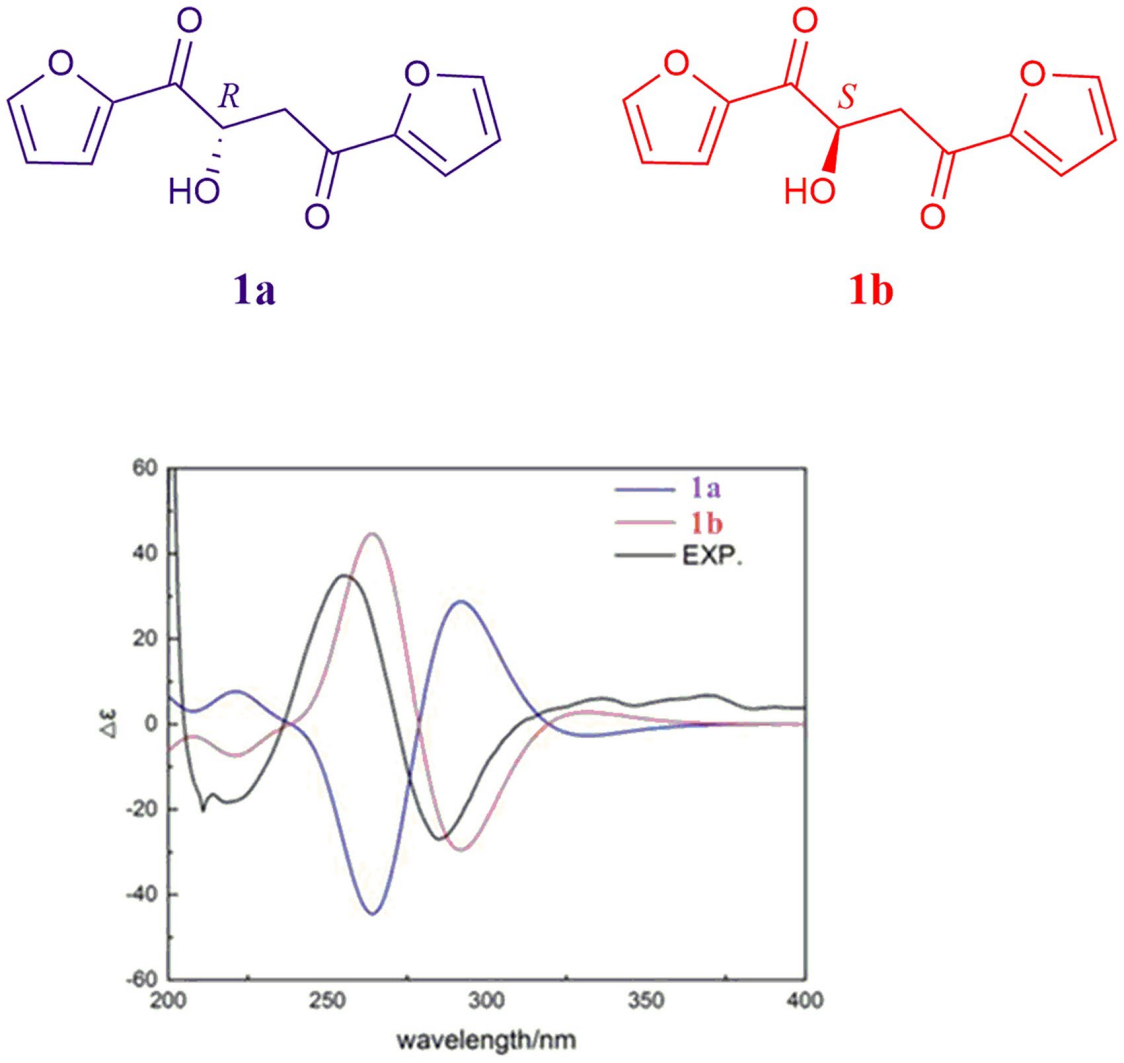
Experimental CD spectrum of **1** and calculated ECD spectra of **1a** and **1b**.

Due to the fact that modified Mosher’s reactions could not determine the absolute configuration of the hydroxyl in **1**, the stereochemistry of **1** was solved by electronic circular dichroism (ECD) combined with quantum-chemical calculations adopting time-dependent density functional theory (Song et al., 2019; Zhang et al., 2021). By comparing the experiment spectrum with the calculated ECD spectra (Figure 6), the stereochemistry of **1** was depicted as in Figure 1.

Four known furanoids were determined to be rhizosolaniol (**2**) (Supplementary Figures S13, S14; Liu et al., 2012), 5-hydroxymethylfurfural (**3**) (Supplementary Figure S15; Li et al., 2012), 2-furoic acid (**4**) (Supplementary Figure S16; Abdel-Lateff et al., 2003) and (2-furyl) oxoacetamide (**5**) (Supplementary Figure S17; Nagaki et al., 2016) based on NMR and HR-ESI-MS data.

### Antioxidant activities

**Figure 7:**
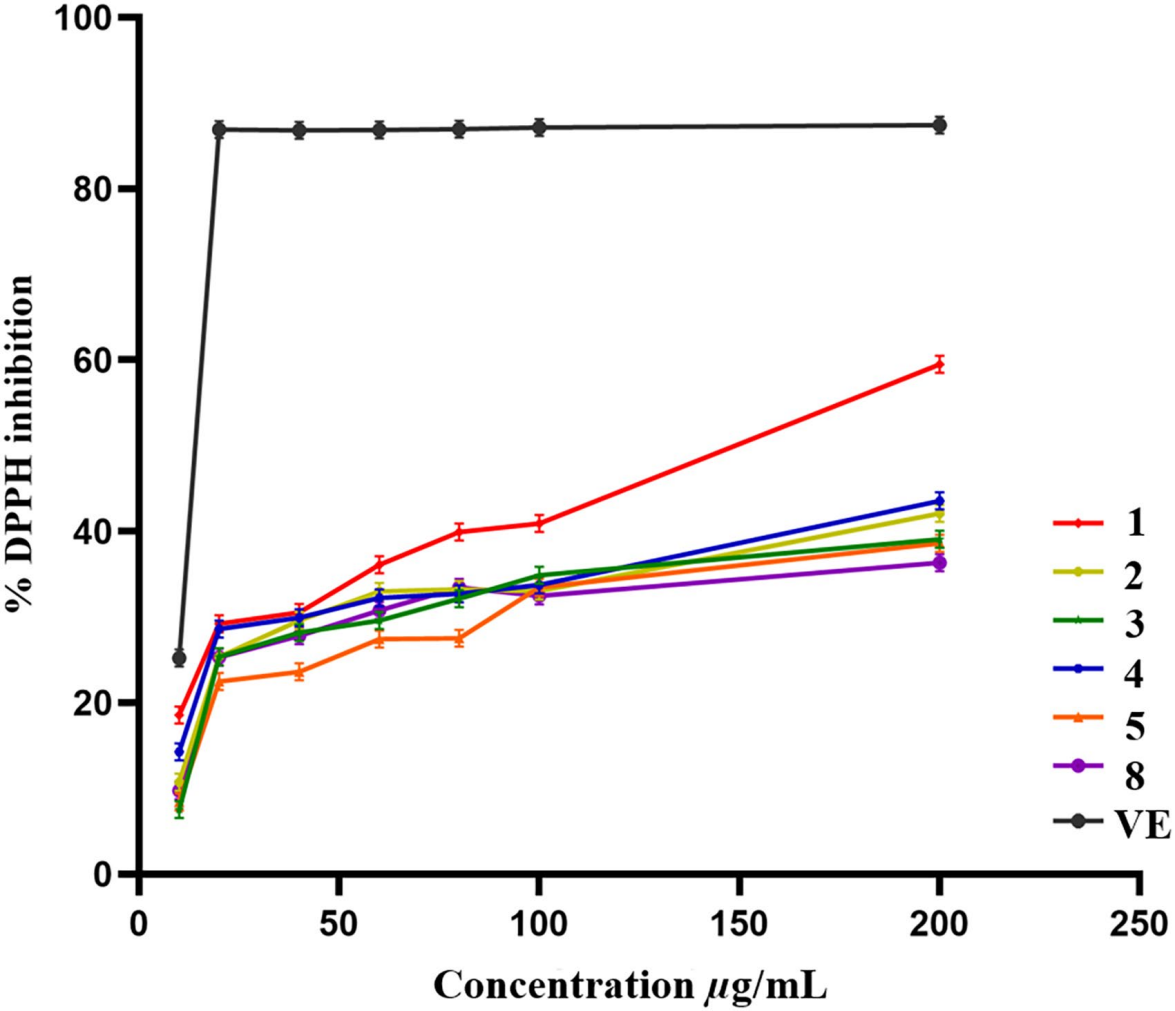
Antioxidant activities of compounds 1-5 and 8.

Compounds **1**–**5** and **8** were evaluated for the antioxidant activities by using DPPH scavenging assay. Compounds **1**–**5** and **8** showed scavenging efficiency with IC_50_ values of 145.5, 381.7, 417.5, 367.2, 480.8, 600.2 μg/ml, respectively, as standard acarbose having IC_50_ value of 13.07 μg/ml (Figure 7).

### Phytotoxicity assay

**Figure 8:**
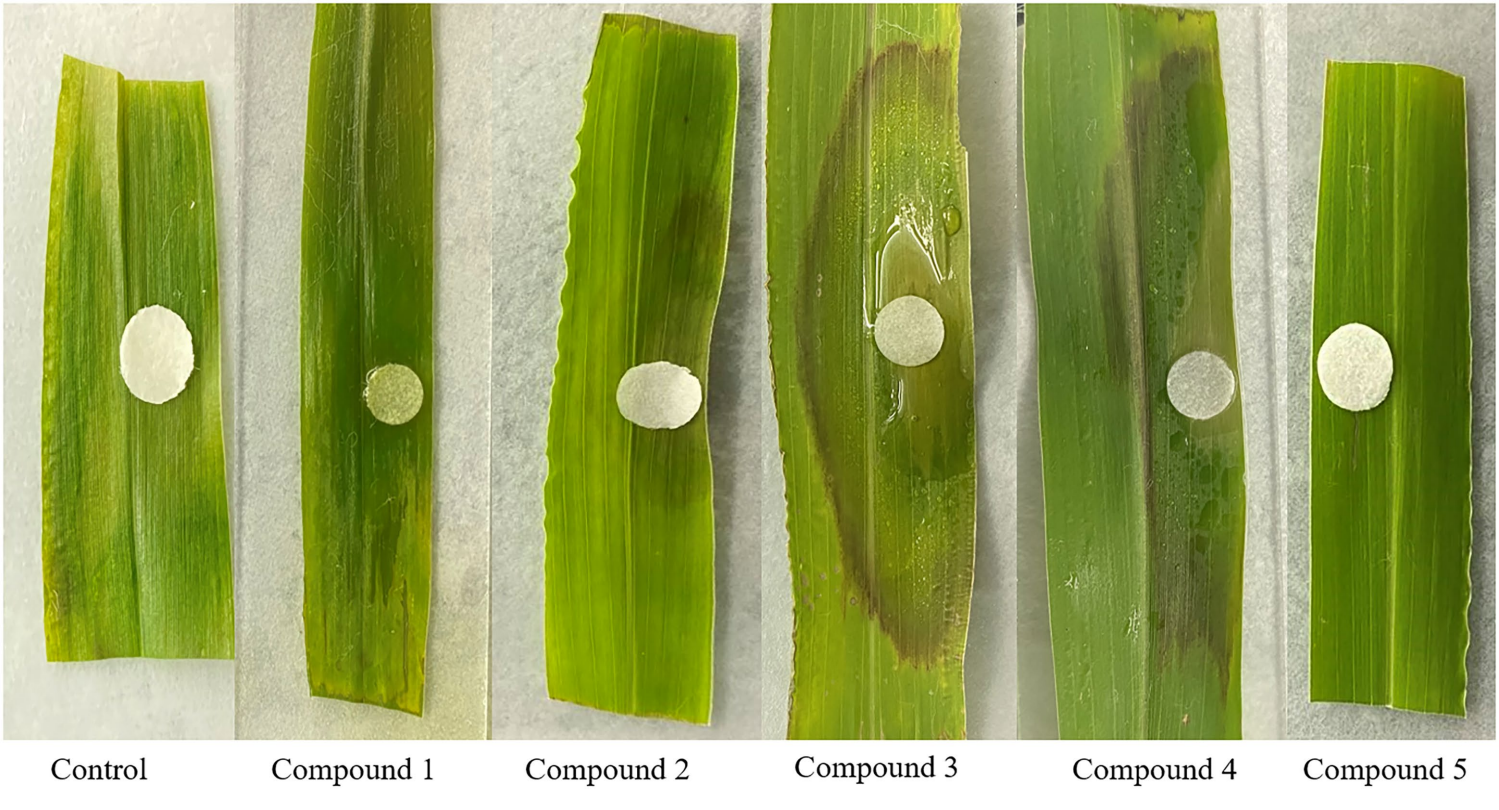
Phytotoxicities of **1**–**5** on the green foxtail leaves.

In the intact detached green foxtail leaves assay, compounds **2**–**4** produced brown spots while compounds **1** and **5** did not produce lesions (Figure 8).

### Germination assay

**Figure 9:**
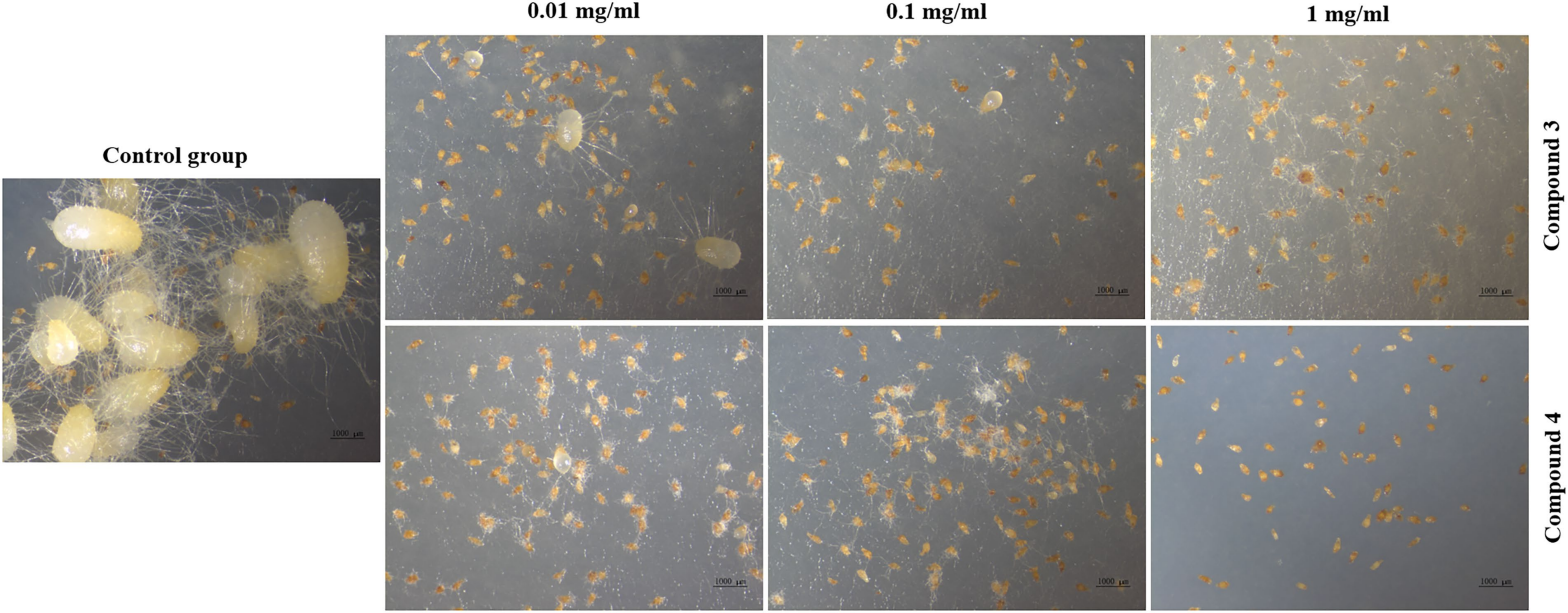
Inhibitory effects of **3** and **4** on seed germination of *G. conopsea*.

**Figure 10:**
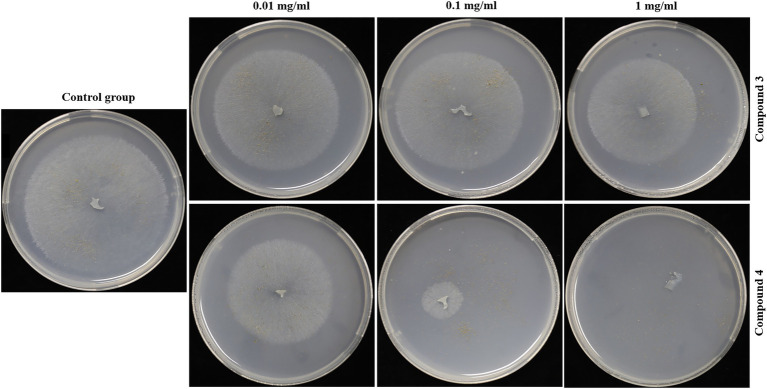
Inhibitory effects of **3** and **4** on GS2.

Seed germination results showed that compounds **3** and **4** could inhibit seed germination of *G. conopsea* (Figure 9), from which the germination rate of all exogenous treatments (**3** and **4**) were lower than the control (14.58%; Supplementary Table S6). Based on observing the growth of the fungus (Figure 10), it revealed that compounds **3** and **4** could inhibit the growth of the symbiotic fungus, which led to the decrease of seed germination rate. Other compounds had not been tested germination assay due to limited amounts in this report.

Symbiotic fungi are able to produce a wide variety of secondary metabolites with complex and unique structures, which make an important role in plant growth and development (Motoyama et al., 2021). Theoretically, GS2 could produce metabolites to promote seed germination. However, in this research, several compounds inhibited the hyphal growth of GS2 itself and led to a lower seed germination rate. The presence of an autoinhibitory substance inhibiting their own growth had been documented in several researches. (Mast and Pace, 1938; Jorgensen, 1956). Additionally, compound **2** isolated from *Rhizoctonia solani* could inhibit seed germination that have also been reported (D’Silva and Herrett, 1971). Previous report suggested that furan organic compounds are of bacteriostatic and fungistatic effect on diverse microorganisms (Vijayakumar and Thirunanasambandham, 2021; Steglińska et al., 2022). Besides, previous studies had confirmed that microbes could produce different secondary metabolites under different culture conditions (D’Silva and Herrett, 1971; Portela et al., 2022). In this research, GS2 was cultured on rice media, whether compounds **1**–**5** or other potential secondary metabolites are capable of promoting seed germination that could be produced under symbiotic condition or on different media deserved to be investigated in future research.

## Conclusion

In this work, a new compound **1** together with four known compounds **2**–**5** were isolated from a symbiotic fungus GS2. Their structures were determined by HR-ESI-MS, NMR (^1^H, ^13^C, ^1^H-^1^H COSY, HSQC, and HMBC spectra) and the absolute configuration of **1** was determined by using quantum chemical electronic circular dichroism (ECD) calculations. Compound **1** was easy to transform into compounds **6**–**9** in Mosher’s reagents which were analyzed using the UPLC-Q-TOF-MS/MS technique in positive mode, from which their possible mass fragmentation patterns were suggested, besides neural loss and 1,3-rearrangement were the main clearage patterns. The bioactive evaluation revealed that compounds **1**–**5** and **8** displayed weak antioxidant activities, and compounds **2**–**4** possessed phytotoxicity against green foxtail leaves. In addition, compounds **3** and **4** displayed remarkable activities of inhibiting seed germination of *G. conopsea*. This was the first chemical investigation of the symbiotic fungus of *G. conopsea*, which could provide potent chemical clue to the symbiotic germination mechanism studies, but more experiments are needed to target the secondary metabolites with the abilities to promote the seed germination of *G. conopsea*.

## Data availability statement

The original contributions presented in the study are included in the article/supplementary material, further inquiries can be directed to the corresponding author.

## Author contributions

XX and GD conceived and designed the study. LS, ZZ, QL, and YW performed the experiments. LS, LH, and GD performed the data collection and analyses. LH performed document processing. LS, GD, and XX wrote the first draft of the manuscript. All authors contributed to the article and approved the submitted version.

## Funding

The work was supported by the National Natural Science Foundation of China (grant no. 32170013) and CAMS Initiative for Innovative Medicine (grant no. 2021-I2M-1-031).

## Conflict of interest

The authors declare that the research was conducted in the absence of any commercial or financial relationships that could be construed as a potential conflict of interest.

## Publisher’s note

All claims expressed in this article are solely those of the authors and do not necessarily represent those of their affiliated organizations, or those of the publisher, the editors and the reviewers. Any product that may be evaluated in this article, or claim that may be made by its manufacturer, is not guaranteed or endorsed by the publisher.
