# Supplementary material for: Furanoids from the Gymnadenia conopsea (Orchidaceae) seed germination supporting fungus Ceratobasidium sp. (GS2)
Source: Front Microbiol. 2022 Nov 17;13:1037292. doi: 10.3389/fmicb.2022.1037292 (PMC9712750; doi:10.3389/fmicb.2022.1037292)
Supplement: Supplementary file 1 [file Data_Sheet_1.docx]

**List of Contents**

**Figure S1**. UV spectrum of compound **1** in MeOH

**Figure S2**. IR spectrum of compound **1** in MeOH

**Figure S3**. HR-ESI-MS spectrum of compound **1** in MeOH

**Figure S4**. ^1^H-NMR spectrum of compound **1** (DMSO-*d*_6_, 600MHz)

**Figure S5**. ^13^C-NMR spectrum of compound **1** (DMSO-*d*_6_, 150MHz)

**Figure S6**. HSQC spectrum of compound **1** (DMSO-*d*_6_, 600MHz)

**Figure S7**. ^1^H-^1^HCOSY spectrum of compound**1** (DMSO-*d*_6_, 600MHz)

**Figure S8**. HMBC spectrum of compound **1** (DMSO-*d*_6_, 600MHz)

**Figure S9.** The ultraviolet absorption of compounds **6** and **8** in MeOH

**Figure S10.** The UPLC-Q-TOF-MS UV of compound **8** in MeOH

**Figure S11**. HR-ESI-MS spectrum of compound **8** in MeOH

**Figure S12.**^1^H-NMR spectrum of compound **8** (CDCl_3_, 150MHz)

**Figure S13**. ^1^H-NMR spectrum of compound **2** ((CD_3_)_2_CO, 600MHz)

**Figure S14**. HR-ESI-MS spectrum of compound **2** in MeOH

**Figure S15**. ^1^H-NMR spectrum of compound **3** (CDCL_3_, 600MHz)

**Figure S16**. ^1^H-NMR spectrum of compound **4** (DMSO: 600MHz)

**Figure S17**.^1^H-NMR spectrum of compound **5** (CDCl_3_, 600MHz)

**Table S1.** Elemental constituents of major product ions from [M + Na]^+^ for **1**

**Table S2.** Elemental constituents of major product ions from [M + H]^+^ for **6**

**Table S3.** Elemental constituents of major product ions from [M + Na]^+^ for **7**

**Table S4.** Elemental constituents of major product ions from [M + Na]^+^ for **8**

**Table S5.** Elemental constituents of major product ions from [M + H]^+^ for **9**

**Table S6.** The seed germination rate of compounds **3**–**4** and the control group on *G. conopsea*


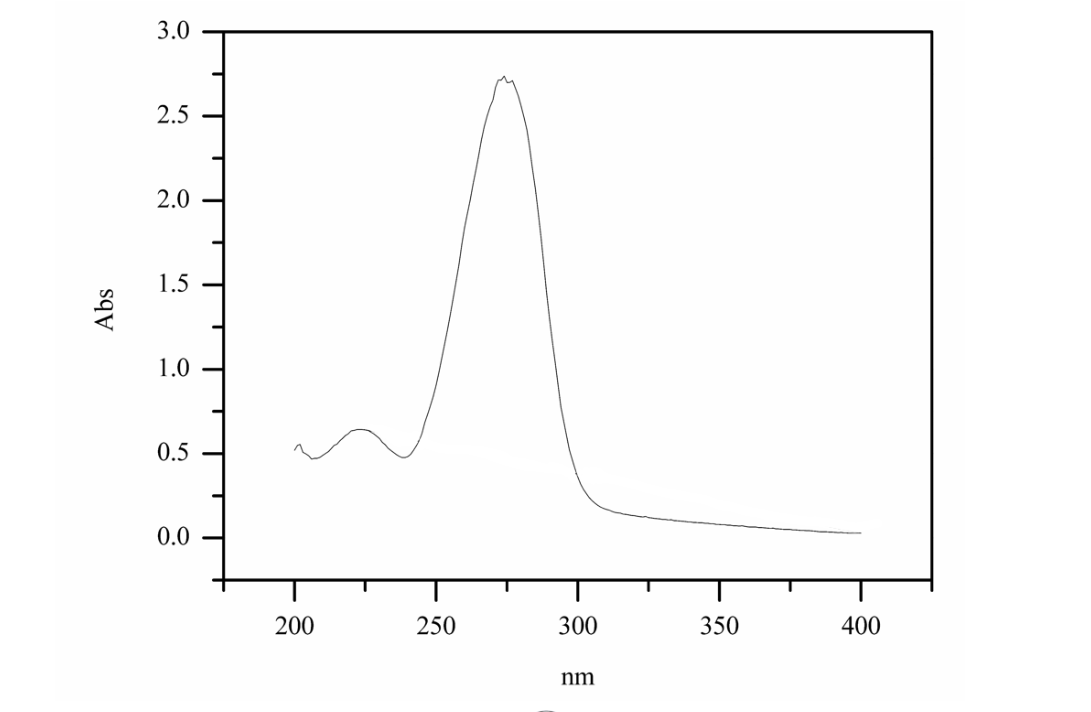


**Figure S1**. UV spectrum of compound **1** in MeOH


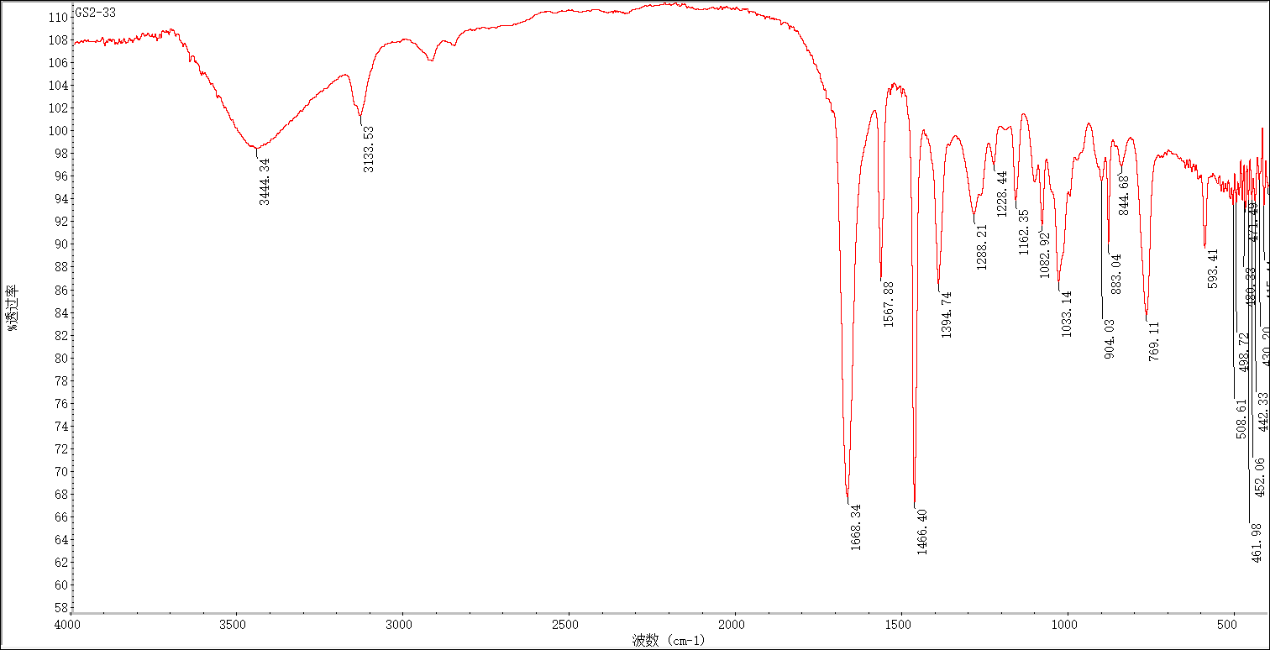


**Figure S2**. IR spectrum of compound **1** in MeOH

**Figure S3**. HR-ESI-MS spectrum of compound **1** in MeOH

**Figure S4**.^1^H-NMR spectrum of compound **1** (DMSO-*d*_6_, 600MHz)

**Figure S5**.^13^C-NMR spectrum of compound **1** (DMSO-*d*_6_, 150MHz)

**Figure S6**. HSQC spectrum of compound **1** (DMSO-*d*_6_, 600MHz)

**Figure S7**.^1^H-^1^H-COSY spectrum of compound **1** (DMSO-*d*_6_, 600MHz)

**Figure S8**. HMBC spectrum of compound **1** (DMSO-*d*_6_, 600MHz)

**Figure S9.** The ultraviolet absorption of compounds **6** and **8** in MeOH

**Figure S10.** The UPLC-Q-TOF-MS UV of compound **8** in MeOH

**Figure S11**. HR-ESI-MS spectrum of compound **8** in MeOH

**Figure S12**.^1^H-NMR spectrum of compound **8** (CDCl_3_, 150MHz)

**Figure S13**.^1^H-NMR spectrum of compound **2** ((CD_3_)_2_CO, 600MHz)

**Figure S14**. HR-ESI-MS spectrum of compound **2** in MeOH

**Figure S15**.^1^H-NMR spectrum of compound **3** (CDCL_3_, 600MHz)

**Figure S16**.^1^H-NMR spectrum of compound **4** (DMSO: 600MHz)

**Figure S17**.^1^H-NMR spectrum of compound **5** (CDCl_3_, 600MHz)

**Table S1.** Elemental constituents of major product ions from [M + Na]^+^ for **1**

| Fragment ion | Formula | Calculated | Observed | Error (PPM) |
| --- | --- | --- | --- | --- |
| [ M + Na]^+^ | C_12_H_10_O_5_Na | 257.0426 | 257.0418 | -3.1 |
| [ M + H - H_2_O]^+^ | C_12_H_9_O_4_ | 217.0501 | 217.0497 | -1.8 |
| [ M + H - CH_2_O_2_]^+^ | C_11_H_9_O_3_ | 189.0552 | 189.0552 | +2.1 |
| [ M + H - C_4_H_6_O_2_]^+^ | C_8_H_5_O_3_ | 149.0239 | 149.0239 | +0.0 |
| [ M + H - C_5_H_6_O_3_]^+^ | C_7_H_5_O_2_ | 121.0290 | 121.0291 | +0.8 |
| [ M + H - C_7_H_8_O_3_]^+^ | C_5_H_3_O_2_ | 95.0133 | 95.0124 | -9.5 |

**Table S2.** Elemental constituents of major product ions from [M + H]^+^ for **6**

| Fragment ion | Formula | Calculated | Observed | Error (PPM) |
| --- | --- | --- | --- | --- |
| [ M + H]^+^ | C_12_H_9_O_4_ | 217.0501 | 217.0502 | +0.5 |
| [ M + H - CO]^+^ | C_11_H_9_O_3_ | 189.0552 | 189.0545 | -3.7 |
| [ M + H - C_4_H_4_O]^+^ | C_8_H_5_O_3_ | 149.0239 | 149.0240 | +0.7 |
| [ M + H - C_5_H_4_O_2_]^+^ | C_7_H_5_O_2_ | 121.0290 | 121.0290 | +0.0 |
| [ M + H - C_7_H_6_O_2_]^+^ | C_5_H_3_O_2_ | 95.0133 | 95.0125 | -8.4 |

**Table S3.** Elemental constituents of major product ions from [M + Na]^+^ for **7**

| Fragment ion | Formula | Calculated | Observed | Error (PPM) |
| --- | --- | --- | --- | --- |
| [ M + Na]^+^ | C_13_H_12_O_5_Na | 271.0582 | 257.0579 | -1.1 |
| [ M + H]^+^ | C_13_H_13_O_5_ | 249.0763 | 249.0749 | -5.6 |
| [ M + H - CH_3_OH]^+^ | C_12_H_9_O_4_ | 217.0501 | 217.0497 | -1.8 |
| [ M + H - C_2_H_4_O_2_]^+^ | C_11_H_9_O_3_ | 189.0552 | 189.0552 | +0.0 |
| [ M + H - C_5_H_8_O_2_]^+^ | C_8_H_5_O_3_ | 149.0239 | 149.0241 | +1.3 |
| [ M + H - C_6_H_8_O_3_]^+^ | C_7_H_5_O_2_ | 121.0290 | 121.0286 | -3.3 |
| [ M + H - C_8_H_10_O_3_]^+^ | C_5_H_3_O_2_ | 95.0133 | 95.0137 | +4.2 |

**Table S4.** Elemental constituents of major product ions from [M + Na]^+^ for **8**

| Fragment ion | Formula | Calculated | Observed | Error (PPM) |
| --- | --- | --- | --- | --- |
| [ M + H]^+^ | C_12_H_9_O_4_ | 217.0501 | 217.0497 | -1.8 |
| [ M + H - CO]^+^ | C_11_H_9_O_3_ | 189.0552 | 189.0553 | +0.5 |
| [ M + H - C_4_H_4_O]^+^ | C_8_H_5_O_3_ | 149.0239 | 149.0238 | -0.7 |
| [ M + H - C_5_H_4_O_2_]^+^ | C_7_H_5_O_2_ | 121.0290 | 121.0284 | -5.0 |
| [ M + H - C_7_H_6_O_2_]^+^ | C_5_H_3_O_2_ | 95.0133 | 95.0133 | +0.0 |

**Table S5.** Elemental constituents of major product ions from [M + H]^+^for **9**

| Fragment ion | Formula | Calculated | Observed | Error (PPM) |
| --- | --- | --- | --- | --- |
| [ M + H]^+^ | C_24_H_17_O_8_ | 433.0923 | 433.0921 | -0.5 |
| [ M + H - C_4_H_4_O]^+^ | C_20_H_13_O_7_ | 365.0661 | 365.0656 | -1.4 |
| [ M + H - C_5_H_4_O_2_]^+^ | C_19_H_13_O_6_ | 337.0712 | 337.0710 | -0.6 |
| [ M + H - C_9_H_8_O_3_]^+^ | C_15_H_9_O_5_ | 269.0450 | 269.0455 | +1.9 |

**Table S6.** The seed germination rate of compounds **3**–**4** and the control group on *G. conopsea*

| Concentration | 0.01 mg/ml | 0.1 mg/ml | 1mg/ml |
| --- | --- | --- | --- |
| Compound **3** | 9.27% | 2.59% | 0% |
| Compound **4** | 1.97% | 0% | 0% |
| Control group | 14.58% | 14.58% | 14.58% |
